# An Ecological Framework for Cancer Communication: Implications for Research

**DOI:** 10.2196/jmir.7.3.e23

**Published:** 2005-07-01

**Authors:** Kevin Patrick, Stephen S Intille, Marion F Zabinski

**Affiliations:** ^2^House_nMassachusetts Institute of TechnologyCambridge, MassachusettsUSA; ^1^Department of Family and Preventive MedicineUniversity of California, San DiegoLa Jolla, CaliforniaUSA

**Keywords:** Internet, cancer communication, ecological momentary assessment, ecological momentary intervention, ubiquitous computing, ecological models, health behavior

## Abstract

The field of cancer communication has undergone a major revolution as a result of the Internet. As recently as the early 1990s, face-to-face, print, and the telephone were the dominant methods of communication between health professionals and individuals in support of the prevention and treatment of cancer. Computer-supported interactive media existed, but this usually required sophisticated computer and video platforms that limited availability. The introduction of point-and-click interfaces for the Internet dramatically improved the ability of non-expert computer users to obtain and publish information electronically on the Web. Demand for Web access has driven computer sales for the home setting and improved the availability, capability, and affordability of desktop computers. New advances in information and computing technologies will lead to similarly dramatic changes in the affordability and accessibility of computers. Computers will move from the desktop into the environment and onto the body. Computers are becoming smaller, faster, more sophisticated, more responsive, less expensive, and—essentially—ubiquitous. Computers are evolving into much more than desktop communication devices. New computers include sensing, monitoring, geospatial tracking, just-in-time knowledge presentation, and a host of other information processes. The challenge for cancer communication researchers is to acknowledge the expanded capability of the Web and to move beyond the approaches to health promotion, behavior change, and communication that emerged during an era when language- and image-based interpersonal and mass communication strategies predominated. Ecological theory has been advanced since the early 1900s to explain the highly complex relationships among individuals, society, organizations, the built and natural environments, and personal and population health and well-being. This paper provides background on ecological theory, advances an Ecological Model of Internet-Based Cancer Communication intended to broaden the vision of potential uses of the Internet for cancer communication, and provides some examples of how such a model might inform future research and development in cancer communication.

## Introduction

The field of cancer communication has undergone a major revolution as a result of the Internet and the World Wide Web. As recently as the early 1990s, paper- and telephone-based platforms were the dominant methods used to exchange information between health professionals and individuals in support of the prevention and treatment of cancer. Interactive media existed, but they usually required sophisticated computer or video platforms that limited availability. The Internet is changing this as it expands in both availability and capability. As described in several other papers in this issue, novel processes of interpersonal communication are mushrooming as a result of the Web, including synchronous and asynchronous one-to-one, one-to-many, and many-to-many approaches.

Gunther Eysenbach's recent comprehensive review of Internet cancer communication identified four broad application areas: (1) communication via e-mail, instant messaging, and voice over Internet protocol; (2) content in the form of the array of multimedia health information available on the Web; (3) community in the form of chat rooms, bulletin board systems, mailing lists, and other forms of groupware; and (4) e-commerce supporting buyers and sellers of cancer-related goods and services [1]. As Eysenbach outlined in the conceptual model in that paper, from a health behavior perspective, these application areas share a common basis as they support intrapersonal and interpersonal needs such as the acquisition of knowledge, shared decision making, social support, and the development of self-efficacy. In this view, the Internet has reduced barriers of time, space, professional distance, and sometimes culture, but it continues to rely heavily on language or visual representations and conscious psychosocial or cognitive processes. While many of these applications have become widely used and favored by those with or at risk of cancer, their ultimate impact on cumulative cancer morbidity, mortality, and related outcomes remain to be seen [1]. An additional question remains: Is the full potential of the Internet and related computing technologies being used to improve cancer outcomes?

## A Potential Reframing of Internet and Cancer Communication

The intent of any cancer communication strategy is to create a favorable effect on one or more of the determinants in the pathway of the cancer continuum—etiology, prevention, early detection, treatment, and post-treatment survivorship. Broadly conceptualized, these effects can be the result of a wide range of activities, such as helping individuals avoid exposure to substances that might place them at risk for cancer, effecting positive change in behaviors that place individuals at risk for cancer, enabling and optimizing cancer therapy, and helping with social and psychological support that is often essential to both decision making and quality of life for individuals diagnosed with cancer.

However, two recent phenomena—one health related and the other technology related—suggest that it may be appropriate to extend the conceptualization of the potential contributions of Internet communication for cancer beyond application areas that depend upon intra- and interpersonal psychosocial and cognitive processes. The first is the increasing recognition among public health and health behavior researchers of the limitations of interventions based solely on individual psychosocial and cognitive theories and processes. The second is the transformation of the Internet from a medium requiring conscious engagement through language or visual-based devices to one that supports a wide array of communication through passive “use” while at the same time becoming ubiquitous. This paper provides background on these two phenomena and proposes a model that integrates them into a research agenda for Internet-enabled cancer communication interventions.

## The Growth of Ecological Models of Health Behavior

While many theories have been used to help explain health behavior, Bandura's social cognitive theory (SCT) [2] has become one of the primary cornerstones of research into the determinants of health behaviors. SCT has served as the basis for a large proportion of individual-level health behavior interventions, including many Internet-based interventions aimed at preventing cancer or optimizing its therapy once diagnosed. SCT suggests reciprocal causation between behavior and intrapersonal and environmental factors. Intrapersonal factors include individual characteristics (eg, age, gender) and cognitions and attitudes about behaviors (eg, self-efficacy, knowledge, perceived benefits). From the perspective of SCT, environmental factors are typically limited to those in the social and cultural environment. As a result, SCT-based intervention research focuses mostly on individual factors and often lacks meaningful evaluation of the potential impact of the full range of environmental determinants of health behavior. This is particularly unfortunate at a time of increasing understanding of both the importance of and the complex relationships among genetic, behavioral, and environmental factors in the causal pathway for cancer.

To address this, some researchers have begun to recognize the limitations inherent in theories based on cognitive processes and have proposed more comprehensive ecological models and theories that are more inclusive of the many environmental factors that may affect health behaviors [3,4]. Ecological theories posit that health and behavior are influenced at multiple levels, including interpersonal, sociocultural, policy, and physical environmental factors, and that these influences interact with one another [5-8]. For example, ecological models include an emphasis on characteristics of the built environment, such as architecture and community design, access to elements important to behaviors such as tobacco and healthy or unhealthy food, opportunities for physical activity, and the impact of technologies such as television or other media. At the largest level, these models and theories recognize the effect of natural environmental factors such as geography, weather, and climate on health behavior [4].

An example of the utility of ecological models may be found in their ability to explain the levels of intervention that have been shown to be necessary to address tobacco use. As outlined in a recent US Surgeon General report, these include clinical intervention, educational efforts, regulatory efforts, economic policies, and combined efforts at all of these levels [9]. No single element in this set of activities is sufficient, but rather it is the synergistic interaction among all levels that results in sustained behavior changes [10]. Increasing recognition of the limitations of intrapersonal, interpersonal, and cognitive interventions for health behavior change is leading researchers in areas such as obesity [11] and physical activity [12,13] to broaden their perspectives on opportunities for research and public health practice. However, the ecological perspective is rarely evident in research and practice in health behavior interventions utilizing the Internet. The historical roots of cancer communication—oral, written, and visual communication—are highly prevalent today. Little evidence is found in the health communication and health behavior literature about how Internet-based technologies might inform and support health promoting interactions with larger environmental processes.

## Ubiquitous Computing

Paralleling the growth of the World Wide Web over the past 15 years has been the related development of ubiquitous computing, first envisioned by Mark Weiser of Xerox PARC as “the idea of integrating computers seamlessly into the world at large” [14]. The prescience of Weiser's vision is remarkable given the current widespread deployment of cell phones, laptops, Wi-Fi, Bluetooth, personal digital assistants (PDAs), and various forms of sensing devices based on digital and radio frequency identification (RFID) technologies. A vast and multilayered infrastructure of ubiquitous computing technologies and applications is emerging. Current functionality predominately supports business and commerce through a myriad of examples, including laptop computers and cell phones with software allowing full-function mobile work; transcontinental tracking of cargo containers with global positioning systems; and RFID-supported inventory and process management systems.

Aboud and Mynatt of Georgia Tech have articulated the challenges for optimizing this “ubicomp” environment as three-fold: (1) developing natural interfaces that facilitate a wide variety of interaction between humans and computational devices; (2) rendering ubiquitous computing devices fully context aware, capable of sensing the physical and natural environments and adapting information gathering and presentation based upon this; and (3) supporting automated capture of experiences in real time to enable subsequent access and use [15]. While their analysis does not specifically address health-related ubicomp research, reports of such work are beginning to appear, including studies that focus on the social interaction needs of elders experiencing age-related cognitive problems [16], hospital-based experiments in context-aware computing [17], and just-in-time dietary behavior intervention [18]. However, an extensive search of the medical (PubMed), psychological (psychINFO), and communication (Communication Abstracts) literatures in July 2004 found no substantive attention to the potential for ubicomp to improve interventions for cancer-related behavior or cancer communication research. Research in the engineering domains has primarily focused on developing new sensors for health monitoring of critical events such as falls or stroke, not on longitudinal evaluation of technologies for health maintenance and well-being.

## An Ecological Model of Cancer Communication and Ubiquitous Computing

**Figure 1:**
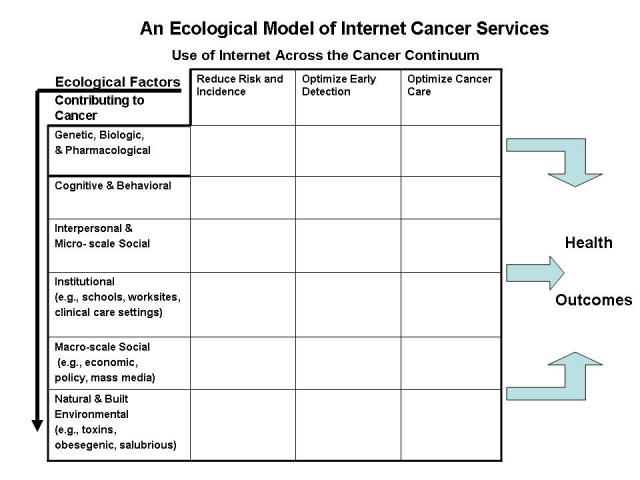
Proposed ecological model for cancer communication

Until now, one of the reasons for the minimal attention given to the potential contributions that ubiquitous computing might make to cancer prevention and control is that there have been no conceptual models advanced that articulate this potential, especially in ways that are grounded in ecological theory. The model proposed in Figure 1 is an attempt to address this deficit.

The horizontal axis portrays the cancer continuum from risk and exposure through detection and then to cancer care. The vertical axis portrays the multiple levels at which cancer risk, disease, and treatment determinants and influences act, from micro-scale biological and intrapersonal factors through social, institutional, and cultural factors, and ultimately to macro-scale built and natural environmental factors. Because ubiquitous computing is not necessarily dependent on conscious intrapersonal and interpersonal processes and may link more to such things as geospatial and object-to-object relationships, these larger ecological levels portray where the potential for ubiquitous computing effects might exist.

**Figure 2:**
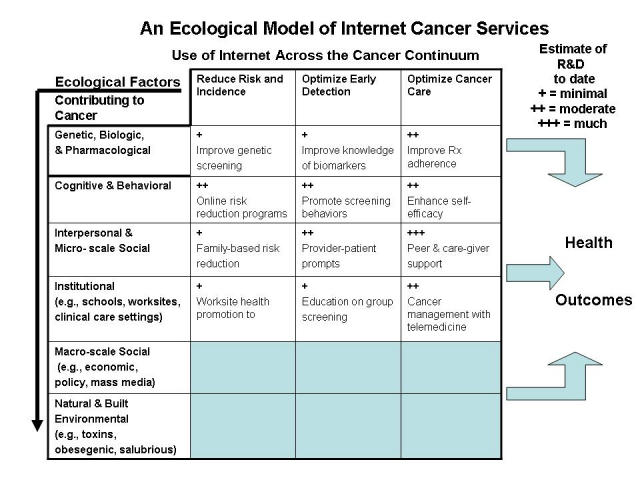
Estimate of progress to date on research of Internet cancer services

As an ecological model, the intent is to be comprehensive and encompass the universe of Internet-based cancer communication interventions. If it accomplishes this, the model can then help inform us about where we have made progress to date and where we have yet to make meaningful impact. While gauging the amount of effort to date is difficult, Figure 2 displays a crude estimate of the level of current research and development in each of these areas. For example, the functions outlined in Eysenbach's recent comprehensive review, communication, content, community, and e-commerce [1], can be apportioned as appropriate into various combinations of cells in the top four rows of the model. In this model, Web-based programs aimed at reducing cancer risk through efforts such as smoking cessation, physical activity promotion, and dietary change would primarily be apportioned to row 2, column 1. Systems deployed by health plans that target—and sometimes tailor—Web-based outreach to enhance mammography utilization can be apportioned to row 4, column 2. Peer-to-peer systems that improve the quality of life of cancer survivors represent activities depicted in row 3, column 3.

As outlined in the papers in this special issue of the Journal of Medical Internet Research, Internet-based systems that support several related functions are gaining in both sophistication and use. But what of the areas in this model that remain relatively unexplored? Are there any traditional Web- or Internet-based interventions that could help fill these gaps? And what is the potential for interventions and applications based on ubiquitous computing to help fill these gaps?

One example of a Web-based desktop application that might be apportioned to row 6, column 1 is a design tool for home customization that could help a homeowner reflect upon the tradeoffs involved as he or she makes home design decisions. Desktop simulation tools are being conceptualized at Massachusetts Institute of Technology (MIT), for example, that help people consider not only immediate concerns such as cost and aesthetics but also long-term implications of design decisions that might impact health and aging in place. Included in row 5, column 1 could be a Web or mobile phone application used by television viewers to instantly register impressions of the content they are watching. Such a system could be used to create a health behavior advertisement that changes in real time based upon who is watching and what opinions the viewers express through the Internet. Widespread distribution of wireless pedometers that attach to shoelaces and automatically send data via the Internet could allow macro-scale monitoring of recovering cancer patients and allow researchers to study correlations between exercise patterns and cancer recovery, an application that would be apportioned to row 5, column 3.

Ubiquitous computing technologies such as wireless communication, sensors, context aware devices, and automated data capture, synthesis, and feedback might contribute to cancer communication in a variety of ways. Initial insight into how this might happen can be seen in the field of ecological momentary assessment (EMA), a method of data collection increasingly used in research that requires the collection of self-reported data on people's experiences as they go about their everyday lives [19,20]. EMA methods have emerged in response to the problems inherent in retrospectively collecting data on such things as mood, pain, and sense of well-being. As these may vary in intensity, duration, and frequency from day to day, hour to hour, or minute to minute based upon ecological context, the validity and reliability of after-the-fact assessments are highly suspect. On the other hand, frequent instantaneous reports of these phenomena have been shown to minimize recall bias and more faithfully represent the true natural history of transitory states.

Systems for EMA were initially developed around paper-based data collection methods. With the advent of technologies such as PDAs, handheld computers, and cell phones, this process of prompting for collection of data and the act of data collection itself have become both less cumbersome and more able to incorporate expert logic to facilitate more complex data gathering needs. For example, an expert-system platform can enable certain responses to questions to automatically present more detailed questioning in situations in which richer detail is needed about a given ecological moment.

It requires only a modest extension of logic to envision how EMA systems might begin to incorporate elements of tailored intervention on the very behaviors they are used to measure. Such “ecological momentary intervention” (EMI) could be capable of providing instantaneous and personalized feedback based on a given measured state—and perhaps based on other environmental data like physical location or other contextual factors like social settings. When viewed from the perspective of the ecological model presented in this paper, a technology that, for example, provides a prompt of self-efficacy to avoid calorie dense food in a fast-food establishment could begin to populate the bottom rows of the model. This example of EMI acts on two levels in the model. First, behavioral risk is influenced at the individual level through some form of communication device that provides a message supporting a psychosocially mediated behavior. Second, the device intervenes at the environmental level as it is linked to a wearable sensor triggered by technologies embedded within the built environment, in this case the restaurant.

What of the potential for ubiquitous computing to help complete other cells in the model? Technologies embedded within the environment might assist with home monitoring and adherence to selected elements of cancer therapy. The CareMedia project at Carnegie Mellon University is exploring video monitoring of residents of skilled nursing facilities to enable the analysis of specific individual activities [21]. Given the growth of the elderly population, the emphasis on aging-in-place, and the epidemiology of cancer and many other diseases in this population, there would be considerable value in systems to help monitor things like medication adherence, diet, physical activity, and other behaviors that improve cancer outcomes and enable independent living [22]. Users of these systems could be families of patients who are geographically separated, lay caregivers who may need extra assurance that they are providing the right types of care, or professional case managers. The central function in common, however, would be the presence of monitoring and prompting systems that help optimize the cancer intervention through sensing and monitoring technologies embedded in the physical environment.

The prevention research community can play an important role in ensuring that ubiquitous technologies already being incorporated into the built environment will be available for cancer-related EMI applications. By proposing, prototyping, and validating innovative approaches *now* that populate each of the boxes in the model, health researchers may spur public and private entities to design digital infrastructures so that they are compatible with end-user applications that promote health and well-being. Businesses are actively developing what could be called “ecological momentary advertising” to exploit ubiquitous computing to encourage consumption, but they will not necessarily design the systems to support health applications unless the public, the government, or the health community provides an incentive to do so. Relatively inexpensive modifications to existing devices, such as digital cash registers that can provide an electronic record of what someone bought or ate, could enable powerful new intervention technologies to be created that tailor information at the micro scale and influence policy at the macro scale. Fortunately, even without active participation from companies, emerging mobile devices will be able to gather some information about the built environment, such as where people are and, to some degree, what they are doing. However, built environments that explicitly provide information to enable proactive health applications will enable applications to acquire and exploit detailed records on health-related behaviors with little or no proactive effort on the part of the end user. Simplicity of use transformed the Internet from an unknown technical novelty into a pervasive global information source and communication mechanism in less than 15 years. Simplicity of use of ubiquitous computing devices could enable emergence of innovative data collection and intervention delivery opportunities in a similarly short period of time. The examples in this paper are meant to be illustrative only and derive from current research on the application of ubiquitous computing to health. As with the history of other technologies, it is impossible to predict the type and extent of future applications of technologies that are themselves undergoing rapid change and evolution. Also, perhaps more than many other areas of health research, what is explored—and how—will be heavily influenced by privacy, confidentiality, and “social” issues [15] such as the security of observation and sensing systems, privacy of any recorded data, and the trust required for coexistence with systems that are always “on” and “in control” of selected aspects of daily life.

## Conclusions

The Internet has mushroomed into a vast and important source of information for individuals with health-related concerns in general and cancer-related concerns in particular. Eysenbach estimates that 39% of persons with cancer use the Internet, and an additional 15% to 20% “use” it indirectly through the support and information it provides to their family and friends [1]. While it is not yet clear whether the net impact of Internet use on cancer outcomes is positive, the general sense is that it is, especially when considerations of quality of life are included. Thus, it is incumbent on cancer researchers to explore how to extend the reach of the Internet to all individuals and all relevant domains of the cancer continuum—prevention, early detection, treatment, survivorship, and end-of-life care. Accomplishing this will require conceptualization of the determinants of each of these phases in the broadest possible sense and may be helped through use of ecological models of health. Such models are particularly relevant for Internet cancer communication research given recent trends in ubiquitous computing and the presence of computing and communication technologies of every scale and in essentially every dimension of everyday life. Ubiquitous computing provides the platform to expand psychosocial and cognitive-based cancer communication interventions to include processes embedded in the larger built and natural environments. In the end, the result may be a seamless and continuous support system that optimizes health outcomes at every stage of the cancer continuum.
